# Contribution of neural cell death to depressive phenotypes of streptozotocin-induced diabetic mice

**DOI:** 10.1242/dmm.016162

**Published:** 2014-04-24

**Authors:** Cheng Chen, Yun Wang, Juan Zhang, Lian Ma, Jiang Gu, Guyu Ho

**Affiliations:** 1 Department of Pediatrics, The Second Affiliated Hospital of Shantou University Medical College, Shantou 515041, China.; 2 Department of Molecular Pathology, Shantou University Medical College, Shantou 515041, China.; 3 Laboratory of Translational Medicine, The Second Affiliated Hospital of Shantou University Medical College, Shantou 515041, China.

**Keywords:** Depression, Diabetes, Comorbidity, Apoptosis, Neurodegeneration, Mitochondria

## Abstract

Major depression disorder (MDD) or depression is highly prevalent in individuals with diabetes, and the depressive symptoms are more severe and less responsive to antidepressant therapies in these patients. The underlying mechanism is little understood. We hypothesized that the pathophysiology of comorbid depression was more complex than that proposed for MDD and that neural cell death played a role in the disease severity. To test this hypothesis, we generated streptozotocin (STZ)-induced diabetic mice. These mice had blood glucose levels threefold above controls and exhibited depressive phenotypes as judged by a battery of behavioral tests, thus confirming the comorbidity in mice. Immunohistological studies showed markedly increased TUNEL-positive cells in the frontal cortex and hippocampus of the comorbid mice, indicating apoptosis. This finding was supported by increased caspase-3 and decreased Bcl-2 proteins in these brain regions. In addition, the serum brain-derived neurotrophic factor (BDNF) level of comorbid mice was reduced compared with controls, further supporting the neurodegenerative change. Mechanistic analyses showed an increased expression of mitochondrial fission genes fission protein 1 (*Fis1*) and dynamin-related protein 1 (*Drp1*), and a decreased expression of mitochondrial fusion genes mitofusin 1 (*Mfn1*), mitofusin 2 (*Mfn2*) and optical atrophy 1 (*Opa1*). Representative assessment of the proteins Drp1 and Mfn2 mirrored the mRNA changes. The data demonstrated that neural cell death was associated with the depressive phenotype of comorbid mice and that a fission-dominant expression of genes and proteins mediating mitochondrial dynamics played a role in the hyperglycemia-induced cell death. The study provides new insight into the disease mechanism and could aid the development of novel therapeutics aimed at providing neuroprotection by modulating mitochondrial dynamics to treat comorbid depression with diabetes.

## INTRODUCTION

Diabetes mellitus is a major global health concern, affecting more than 346 million people worldwide (Shrivastava et al., 2013). The hallmark of diabetes is hyperglycemia, and chronic hyperglycemia leads to disabling diabetic complications. Depression claims equal toll on health and economy (Moussavi et al., 2007). It is often comorbid with diabetes, affecting ~20% of the diabetic patient population (Ali et al., 2006; Barnard et al., 2006). There is a malignant interaction between diabetes and depression. Diabetic patients with comorbid depression have poorer glycemic control and a higher incidence of diabetic complications (Egede, 2006; Iosifescu, 2007). Conversely, depression comorbid with diabetes presents with more severe symptoms, has higher rates of relapse and lower rates of remission, and is less responsive to current antidepressant therapies (Regen et al., 2005; Iosifescu, 2007).

Despite the prevalence and severity of comorbid depression in diabetic patients, the underlying mechanisms are little explored, presumably owing to the wealth of research on MDD, for which postulated mechanisms could be applicable to the comorbid depression. There are a number of prevailing molecular hypotheses on MDD. The historical monoamine hypothesis postulates a decreased monoamine function in the brain (Krishnan and Nestler, 2008); the neurotrophic hypothesis proposes a decrement of brain-derived neurotrophic factor (BDNF), a neurotrophic factor that is key to modulating neuroplasticity in the adult brain (Martinowich et al., 2007); the neurogenic hypothesis invokes a need of new neurons in the adult brain for proper mood control (Eisch and Petrik, 2012); the cortisol hypothesis provides a molecular basis for stress-induced depression, where a dysregulated hypothalamic-pituitary-adrenal axis caused either by psychological impact or disease produces excess glucocorticoids, which have been shown to attenuate neurogenesis and promote apoptosis (Zunszain et al., 2011). These proposed molecular disturbances are thought to ultimately alter synaptic plasticity in the hippocampus and cortex, the brain areas thought to be important in regulating affective behaviors (Martinowich et al., 2007; Krishnan and Nestler, 2008; Zunszain et al., 2011; Eisch and Petrik, 2012). In rodent models of streptozotocin (STZ)-induced or db/db diabetes, depressive behaviors have been observed (Miyata et al., 2005; Hirano et al., 2007; Sharma et al., 2010; Ho et al., 2012). A recent study linked the depressive behavior to increased glucocorticoid production, reduced brain BDNF levels and depressed neurogenesis in STZ-induced diabetic mice (Ho et al., 2012), concurring with the theories proposed for MDD.

Whether neural cell death plays a role in MDD is controversial because there is no major cell loss or neuropathology found in postmortem tissues of MDD patients (Czéh and Lucassen, 2007; Krishnan and Nestler, 2008). However, circumstantial evidence suggests that the pathophysiology of depression comorbid with diabetes could be more severe than those proposed for MDD. First, it is well established that glucose overload imposes oxidative stress to cells and that chronic hyperglycemia leads to various organ damages. Second, large-scale and longitudinal studies have shown that diabetes increases the rate of developing dementia (Biessels et al., 2006; van Harten et al., 2007), implicating hyperglycemia in neurodegeneration. Third, in the rodent model of diabetes, a broad range of neural degenerative changes are found by structural and ultrastructural analyses (Hernández-Fonseca et al., 2009). These observations, coupled with more severe depressive symptoms and less treatment response to antidepressants seen in the comorbid patients, led us to hypothesize that, in addition to the relatively subtle molecular and cellular change proposed for MDD, neural cell death in the hippocampus and cortex plays a role in the disease pathology.

**TRANSLATIONAL IMPACT**

**Clinical issue**

Approximately 20% of individuals with diabetes are diagnosed with depression. The depressive symptoms in diabetic patients are often severe and are inadequately treated using current antidepressant medications. The underlying mechanisms of diabetes-associated depression are not understood, hampering efforts at improved intervention. There is some evidence that the pathophysiology of depression that is comorbid with diabetes could be more complex than that of clinically diagnosed depression [known as major depressive disorder (MDD)]. A hypothesis that has emerged is that neural cell death plays a role in the severity of comorbid depression, but this remains to be tested.

**Results**

Here, the authors used behavioral analyses in a mouse model of diabetes to demonstrate the presence of depression-like behaviors in association with the metabolic disorder. They used this model to test whether neural cell death, not generally considered to underlie MDD, contributes to depression in the comorbid mice. They applied immunohistochemical and biochemical approaches to analyze cellular and molecular changes indicative of cell death in the hippocampus and frontal cortex of the mouse brain; these areas are thought to be crucial in mood control. They show that neural cell death is associated with the depressive phenotype of comorbid mice and that hyperglycemia-induced mitochondrial dysfunction plays a role in the cell death observed.

**Implications and future directions**

These findings suggest that neural cell death, particularly affecting the hippocampus and frontal cortex, might be part of the brain pathology underlying depression that is concurrent with diabetes. This conclusion is supported by the high prevalence of depression in Alzheimer’s disease, of which neural cell death is a hallmark feature. The mechanistic insights provided by this study might have implications for the development of neural-protective therapeutic agents to treat depression in diabetes.

Mitochondria support multiple aspects of cell functions by providing cellular energy, generating reactive oxygen species (ROS) and regulating apoptosis (Davis and Williams, 2012). Accumulating evidence suggests that dysregulated mitochondrial dynamics between fission and fusion plays a key role in the pathogenesis of neurodegenerative diseases (Davis and Williams, 2012). Mitochondrial fusion mediated by the GTPases Mfn1, Mfn2 and Opa1 allows the exchange of mitochondrial contents between neighboring mitochondria to promote survival, and fission mediated by Drp1 and Fis1 regulates apoptosis through segregation of critically injured mitochondria (Ranieri et al., 2013). Mutations resulting in fission-dominant expression of genes involved in mitochondrial dynamics have been implicated in a broad range of neurodegenerative diseases, including Alzheimer’s disease (AD), Huntington’s disease (HD) and Parkinson’s disease (PD) as well as some rare forms of neurodegenerative diseases (Itoh et al., 2013; Ranieri et al., 2013). Supporting the clinical findings, *in vitro* studies have demonstrated that overexpression of mitochondrial fission genes leads to mitochondrial fragmentation and apoptosis, whereas overexpression of fusion genes protects cells against apoptosis (Itoh et al., 2013; Ranieri et al., 2013). *In vitro* studies have also shown that, under high-glucose culture conditions, primary cells such as dorsal root ganglia, coronary endothelial cells, cardiovascular cells and pancreatic β-cell lines exhibit a fission-dominant gene expression, resulting in mitochondrial fission and apoptosis (Yu et al., 2008; Men et al., 2009; Makino et al., 2010; Vincent et al., 2010). Therefore, we surmised that a dysregulated expression of genes involved in mitochondrial dynamics could play a role in a hyperglycemia-induced neural cell death *in vivo*.

To test these hypotheses, we generated STZ-induced diabetic mice. The diabetic conditions and depressive behaviors of STZ-treated mice were confirmed. Immunohistochemical and biochemical analyses showed markedly increased apoptotic events in the hippocampus and frontal cortex of comorbid mice and the apoptosis was associated with a fission-dominant expression of genes and proteins mediating mitochondrial dynamics. The data demonstrated for the first time that apoptosis contributed to the depressive phenotype of diabetic mice and that a dysregulated expression of genes and proteins mediating mitochondrial dynamics played a role in the hyperglycemia-induced neural cell death.

## RESULTS

### Generation of STZ-induced diabetic mice

**Fig. 1.**
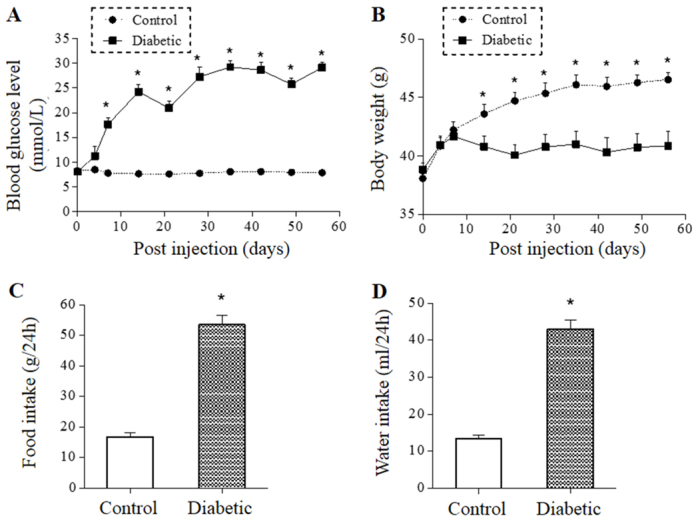
**Generation of STZ-induced diabetic mice.** Mice were administered with STZ and blood glucose levels (A) and body weight gains (B) were measured dynamically for 8 weeks post-STZ treatment. Food and water intakes were measured on day 38 (C,D). Asterisks (*) denote statistical differences (*P*<0.05) between the STZ-treated (*n*=8) and control (*n*=10) groups.

Mice were administered STZ, which selectively destroys β-cells by DNA methylation (Lenzen, 2008), thus producing insulin-deficient diabetic mice. Blood glucose and body weight of mice were monitored dynamically for 8 weeks (56 days) after the STZ treatment. As shown in Fig. 1A, the blood glucose level of control mice remained at ~8 mmol/l throughout the study course, whereas the blood glucose level of STZ-treated mice rose to ~24 mmol/l over the course of 2 weeks and stayed elevated in the range of 22–28 mmol/l (averaged ~threefold above the control) for the remaining 6 weeks of the study. The body-weight gain of STZ-treated mice stopped at day 7 after the STZ treatment and stayed at 41 grams on day 56, whereas the control mice continued to gain weight, reaching 46 grams at the completion of the study (Fig. 1B). The 24-hour food and water intakes, measured on day 38, when the blood glucose and body weight had reached the steady state, were both increased by threefold in the diabetic mice compared with controls (Fig. 1C,D). Thus, the STZ-treated mice exhibited the classic insulin-deficient diabetic signs of hyperglycemia, polydipsia, polyphagia and weight loss.

### Behavioral analyses of diabetic mice

**Fig. 2.**
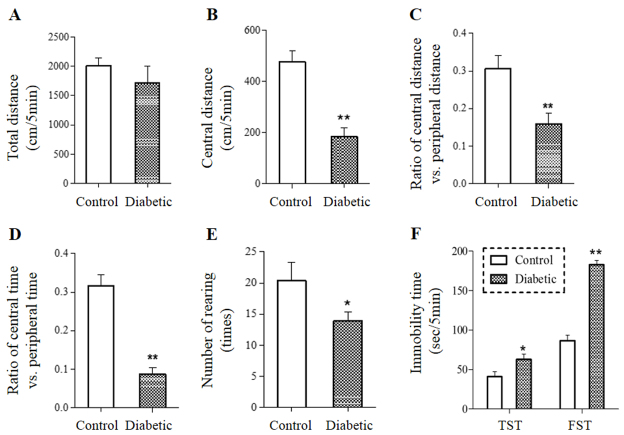
**Behavioral test parameters of diabetic mice.** Diabetic (*n*=8) and control (*n*=10) mice were subjected to a 5-minute OFT. Parameters on the total distance traveled (A), central distance traveled (B), ratio of distance traveled in the center over that in the periphery (C), ratio of time spent in the center over that in the periphery (D) and number of rearings (E) were analyzed. Mice were also subjected to a 5-minute TST and FST, and the immobility time was assessed (F). *P<0.05, **P<0.01.

Anxiety is a common symptom associated with depression. Thus, STZ-treated mice were first subjected to the open-field test (OFT) in the eighth week after the STZ treatment. OFT assesses the anxiety-like behavior as well as the general locomotor activity of rodents. As shown in Fig. 2A, the total distances traveled between the diabetic mice (1700±300 cm) and control mice (2000±120 cm) in the 5-minute test section were not statistically different, suggesting that diabetic mice did not have an apparent deficit in the motor function. However, diabetic mice exhibited a 60% reduction in the distance traveled in the central area of the test field (Fig. 2B), resulting in a 50% reduction in the ratio of the distance traveled in the central area over that traveled in the periphery (i.e. the total distance – central distance) (Fig. 2C). Diabetic mice also spent less time in the central area (24±4 seconds) than the control (71±5 seconds) (Fig. 2D). In addition, diabetic mice displayed a fewer number of times rearing (14±1) than the control (20±3) (Fig. 2E). These data suggest that the diabetic mice, although showing no apparent motor deficit, were less interested in exploratory activities.

Depressive phenotypes of the diabetic mice were evaluated in the eighth week of the study by tail-suspension test (TST) and forced swimming test (FST), the most widely used assays to assess antidepressant efficacy in preclinical studies (Krishnan and Nestler, 2011). TST or FST immobility has been interpreted as an expression of behavioral despair. When subjected to the two tests, diabetic mice showed a respective 55% and 110% increase in the immobility time in TST and FST as compared with controls (Fig. 2F).

### Analysis of apoptosis in the hippocampus and frontal cortex of comorbid mice

**Fig. 3.**
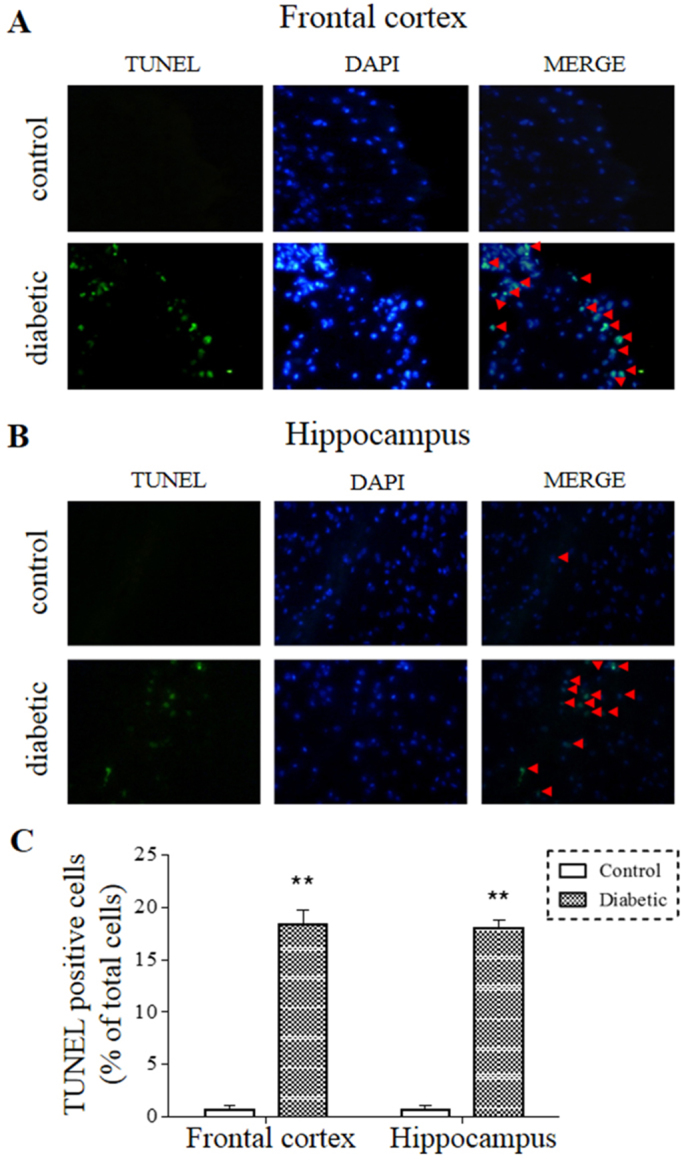
**TUNEL-positive cells in the brain of comorbid mice.** The brain sections were TUNEL stained and counterstained with DAPI. Representative photomicrographs from the frontal cortex (A) and hippocampal CA2 area (B) of TUNEL staining, DAPI staining, and merged TUNEL and DAPI staining are shown. Apoptotic cells are indicated by red arrowheads. Quantification (C) was performed on eight sections/frontal cortex or hippocampus (*n*=6 mice/group). Magnification at 40× objective field. **P<0.01.

Hippocampal and cortical regions of the brain are thought to play an important role in regulating affective behaviors and have been the focus of depression research (Krishnan and Nestler, 2008). To investigate potential apoptotic events in these brain areas of diabetic mice, animals were sacrificed following the behavioral tests and the TUNEL assay was performed in brain tissues to examine the nucleosomal DNA breakage. TUNEL-positive cells were seen throughout the frontal cortex and hippocampus of comorbid mice, whereas very few TUNEL-positive cells were seen in the corresponding regions of control mice (Fig. 3A,B). Quantitative analyses showed that comorbid mice had 18±2% TUNEL-positive cells in the cortex and 17±1% in the hippocampus, as compared with ~1% in the corresponding regions of control mice (Fig. 3C).

**Fig. 4.**
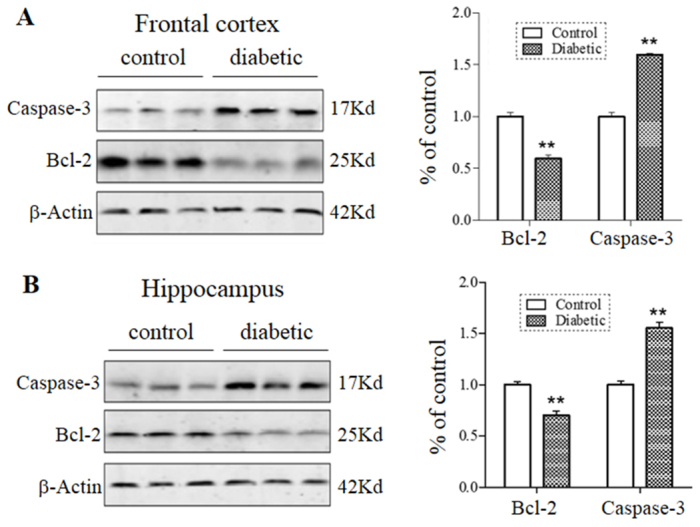
**Caspase-3 and Bcl-2 protein levels in the brain of comorbid mice.** Tissue homogenates of the frontal cortex (A) and hippocampus (B) from comorbid and control mice (*n*=8/group) were analyzed for caspase-3 and Bcl-2 protein levels by western blots. Representative blots are shown with β-actin as internal controls. Quantification was normalized against β-actin. **P<0.01.

To support the immunohistochemical data, the proapoptotic protein caspase-3 (cleaved form) and anti-apoptotic protein Bcl-2 were analyzed by western blots. As shown in the representative blots (Fig. 4A,B), there was an apparent increase of caspase-3 and decrease of Bcl-2 protein levels in both the frontal cortex and hippocampus in diabetic mice. Quantification by densitometric scans indicated that caspase-3 protein levels were increased by ~60% in the two brain regions of comorbid mice, whereas Bcl-2 protein levels were decreased by a respective 30% and 45% in the cortex and hippocampus as compared to the control (Fig. 4). Thus, the biochemical data supported the TUNEL result and indicated that significant apoptosis occurred in the hippocampus and cortex of comorbid mice.

### Analysis of serum BDNF levels

**Fig. 5.**
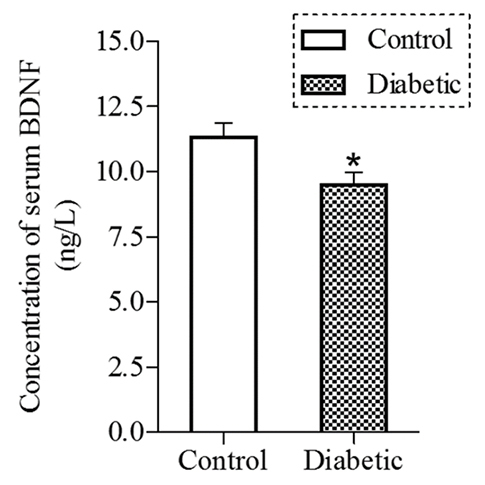
**Serum BDNF levels of comorbid mice.** The terminal blood was collected from comorbid (*n*=8) and control (*n*=10) mice and analyzed for BDNF levels by ELISA. *P<0.05.

BDNF is abundantly expressed in the CNS and is one of the key molecules modulating brain plasticity. Studies have shown altered serum BDNF levels in a variety of neurodegenerative diseases such as AD and PD as well as in mood disorders such as depression (Teixeira et al., 2010). Correlation studies across species have established the direct relationship between BDNF levels in the brain and serum (Klein et al., 2011). To ascertain whether there was a change in the BDNF level of comorbid mice, the terminal blood was assessed by Elisa. As shown in Fig. 5, there was a 15% reduction of the serum BDNF level in comorbid mice as compared with the control.

### Analysis of the expression of genes and proteins mediating mitochondrial dynamics

**Fig. 6.**
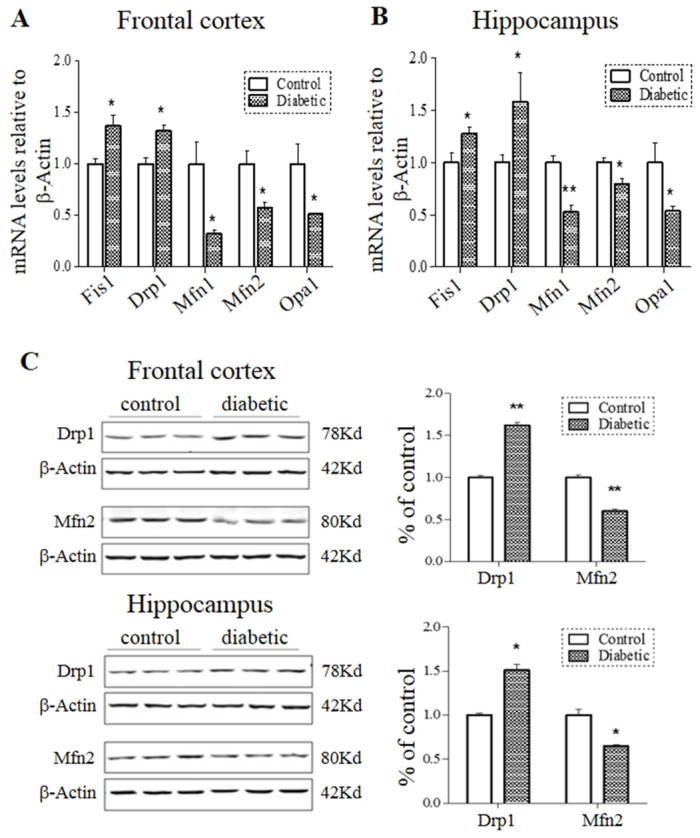
**Expression of genes and proteins mediating mitochondrial dynamics in the brain of comorbid mice.** mRNAs levels of mitochondrial fusion genes *Mfn1*, *Mfn2* and *Opa1* and fission genes *Fis1* and *Drp1* from comorbid and control mice (*n*=8/group) were evaluated by qPCR using β-actin as internal controls (A,B). Protein levels of Mfn2 and Drp1 were analyzed by western blots (C). Representative blots are shown with β-actin as internal controls. Quantification was normalized against β-actin. *P<0.05, **P<0.01.

To investigate whether a dysregulated expression of genes governing mitochondrial dynamics played a role in the observed neural cell death, mRNAs were isolated from the frontal cortex and hippocampus of mice and expression of the mitochondrial fusion genes *Mfn1*, *Mfn* and *Opa1* and the fission genes *Fis1* and *Drp1* were analyzed by qPCR. As shown in Fig. 6A,B, expression of the three fusion genes was reduced by 22–70% in the hippocampus and frontal cortex of comorbid mice, whereas expression of the two fission genes were increased by 25–60% in the two brain regions, compared with controls. To assess whether the mRNA change was translated into protein changes, representative fusion protein Mfn2 and fission protein Drp1 were examined by western blots. As shown in Fig. 6C, increased Drp1 and decreased Mfn2 protein levels were found in both the frontal cortex and hippocampus. Quantification by densitometric scans indicated that Drp1 proteins were increased by ~50% and Mfn2 proteins were reduced by ~40% in the two brain regions. Thus, the data demonstrated a fission-dominant expression of genes and proteins mediating mitochondrial dynamics in the comorbid mouse brain.

### Analysis of association between apoptosis and behavioral impairment in comorbid mice

To establish a causal connection between apoptotic events and depression and/or anxiety-like behaviors in comorbid mice, we performed correlation analyses between the apoptotic parameters of TUNEL staining, caspase-3 levels and Bcl-2 levels in the hippocampus and frontal cortex and behavioral test parameters generated from OFT, TST and FST using the Spearman’s rank test. Of the 36 sets of bivariate correlation analyses (Table 1), 33 showed either strong positive correlations, with *r* values of 0.603 to 0.873 (*P*<0.05), or strong negative correlations, with *r* values of −0.506 to −0.824 (*P*<0.05). The other three correlations, analyzed between mouse rearing in OFT and hippocampal Bcl-2, cortical Bcl-2 or cortical caspase-3 levels, displayed a fair *r* value of 0.476, 0.453 and −0.450, respectively, but they did not reach statistical significance (*P*>0.05). Although a larger sample size could improve the statistical power, the rearing parameter was significantly correlated with TUNEL staining in the hippocampus and cortex as well as with hippocampal caspase-3 levels. Thus, the correlation analyses supported the causal association between brain apoptosis and behavioral abnormalities in comorbid mice.

**Table 1.**
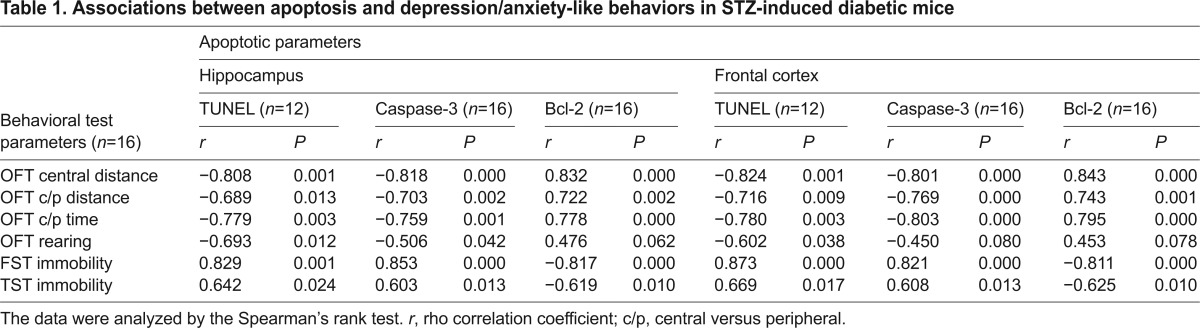
Associations between apoptosis and depression/anxiety-like behaviors in STZ-induced diabetic mice

## DISCUSSION

In this study, we generated STZ-induced diabetic mice that displayed depressive phenotypes. We showed that the comorbid mice exhibited markedly increased apoptosis in the hippocampus and frontal cortex, and that apoptosis was associated with a fission-dominant expression of genes and proteins mediating mitochondrial dynamics. These findings could have broad mechanistic and therapeutic implications in humans.

STZ-induced diabetes is a well-established rodent diabetic model. In our study, STZ treatment resulted in an ~threefold rise in blood glucose, which was maintained throughout the study course. The STZ-treated mice exhibited classic diabetic signs of weight loss, hyperphagia and polydipia (Fig. 1), confirming the establishment of diabetic conditions. When assessed for behavioral abnormalities in the eighth week post-STZ treatment, diabetic mice exhibited significantly reduced exploratory activities measured by OFT (Fig. 2B–E) as well as behavioral despairs measured by prolonged immobility times in TST and FST (Fig. 2F). Because the diabetic mice displayed no apparent motor deficit (Fig. 2A), the reduced exploratory activity was unlikely to be a result of a diabetic peripheral neuropathy. Thus, the comorbid mouse model generated supports and extends previous reports showing STZ-induced diabetic rodents with impaired TST (Miyata et al., 2005; Hirano et al., 2007; Ho et al., 2012). Of note, other reports have shown that STZ-induced diabetic rodents exhibit reduced locomotor activity (Grzeda et al., 2007; Hirano et al., 2007; Ho et al., 2012). The discrepancy could be due to the higher (3.5- to 5-fold above the control) and longer (up to 12 weeks after STZ treatment) glycemic exposure in the other studies. Regardless, none of the studies associate the reduced locomotor activity to peripheral nerve damage and all ascribe it to a behavioral impairment (Grzeda et al., 2007; Hirano et al., 2007; Ho et al., 2012).

Brain imaging studies have documented volume reductions in the hippocampus and other forebrain regions of MDD patients (Videbech and Ravnkilde, 2004; Savitz and Drevets, 2009). Although neural cell death is an obvious contributor to the volume shrinkage, there is no apparent cell loss found in postmortem brain tissues of depressed subjects (Czéh and Lucassen, 2007). Thus, MDD is mostly considered as a disorder of neuroplasticity and cellular resilience but not a neurodegenerative disease (Czéh and Lucassen, 2007). In this study, we observed markedly more TUNEL-positive cells in the hippocampus and frontal cortex of comorbid mice (Fig. 3), which was supported by an increased abundance of cleaved caspase-3, an enzyme involved in the execution of apoptosis, and a decreased abundance of anti-apoptotic protein Bcl-2, the Bcl-2 family member involved in the initiation of apoptosis (Fig. 4). However, we could not distinguish the cell death between neurons and glia in these studies. Considering that both neural cell types are sensitive to glucose overload (Takahashi et al., 2012), it is likely that they both were undergoing apoptosis induced by hyperglycemic conditions. Interestingly, emerging evidence has implicated glial abnormalities in depression as well (Sanacora and Banasr, 2013).

Brain imaging studies have shown that individuals comorbid with depression and diabetes exhibit a decreased gray matter density in the prefrontal cortex that correlates with impaired executive functioning (Kumar et al., 2008; Watari et al., 2008), implying a degenerative change. Although there are no postmortem studies available yet, our finding of neural cell death in comorbid depressive and diabetic mice would support the speculation that neural cell loss underlies the gray matter density change observed in clinical studies. It is noteworthy that depression is highly prevalent in individuals with AD (Starkstein and Mizrahi, 2006), where neural cell death is characteristic of the disorder. Furthermore, clinical evidence thus far has not provided an efficacious support to use current antidepressants (thought to work via improving synaptic transmission and neurogenesis) in mitigating depressive symptoms of individuals with AD (Sepehry et al., 2012). Our present finding of neurodegenerative changes occurring in the comorbid mice would suggest a utility of new therapeutic modalities aiming at neuroprotection to treat depression comorbid with diabetes.

We showed a reduced serum BDNF level in the comorbid mice. Because prior studies have established a direct correlation between the brain and serum BDNF levels in various species (Klein et al., 2011), the reduced serum BDNF level found in this study would reflect a reduction in the levels of BDNF in the brain of comorbid mice. Consistent with this assumption, reduced brain BDNF levels have been reported in STZ-induced diabetic mice with impaired TST (Ho et al., 2012). The reduced BDNF could be attributed to the increased glucocorticoids in STZ-induced diabetic mice (Stranahan et al., 2008; Ho et al., 2012) because glucocorticoids downregulate the BDNF expression by neural cells (Suri and Vaidya, 2013). The reduced BDNF level found in this study could also be attributed to the neural cell death. Studies have shown that individuals with AD exhibit a stage-dependent reduction of serum BDNF levels, with reductions significantly correlating with the cognitive decline (Laske et al., 2006). In contrast, a recent meta-analysis negates the association between serum BDNF concentrations and the symptom severity of MDD (Molendijk et al., 2013). Whether the serum BDNF value of individuals with depression comorbid with diabetes varies with the severity of depressive symptoms, possibly as a biomarker of chronic hyperglycemia-induced neural cell death, would be worth investigating.

Our study showed a fission-dominant expression of mitochondrial dynamic genes and proteins in the diabetic animal brain. Interestingly, increased levels of the fission proteins Drp1 and Fis1 and decreased fusion proteins Mfn1, Mfn2 and Opa1 are found in individuals with AD (Manczak et al., 2011), where Fis1 and Drp1 display aberrant interactions with the AD-related proteins β-amyloid and hyperphosphorylated tau (Manczak and Reddy, 2012). Increased Drp1 and decreased Mfn1, Mfn2 and Opa1 are also found in the striatum and cortex of individuals with HD (Shirendeb et al., 2012). It is thought that the HD-related protein mutant huntingtin directly binds to Drp1 and stimulates its GTPase activity, leading to excessive mitochondrial fragmentation and neural cell death (Song et al., 2011; Shirendeb et al., 2012). How the hyperglycemia-induced dysregulation of mitochondrial dynamics observed in this study causes neural cell death is presently speculative. Excess mitochondrial ROS production, the major culprit of hyperglycemic complications and organ injury, could be a plausible cause. *In vitro* studies have shown that excess intracellular ROS is associated with an imbalanced expression of mitochondrial dynamic genes/proteins, mitochondrial fragmentation and cell death (Jendrach et al., 2008; Liot et al., 2009; Makino et al., 2010). ROS could also directly or indirectly attenuate several key cellular pro-survival pathways (e.g. PI3K-AKT, JAK2-STAT3), resulting in altered production of Bcl-2 and caspase-3 (Xi et al., 2010; Ansley and Wang, 2013). The Bcl-2 family members have been found in association with the fission machineries (Cassidy-Stone et al., 2008; Montessuit et al., 2010; Brooks et al., 2011). The molecular details connecting ROS, mitochondrial fission and apoptotic activation would await future studies.

Although it is commonly accepted that hyperglycemia is the main contributing factor to the pathogenesis of diabetic complications, studies have implicated the brain insulin resistance or defective brain insulin signaling in neurodegenerative diseases (Bassil et al., 2014). We could not rule out the role of insulin deficiency in the neural pathology observed in the current STZ-induced diabetic model. Brain- or neural-specific insulin receptor knockout (BIRKO) mice, which have normal blood glucose levels but are deficient in the brain insulin signaling, display no apparent brain morphological or behavioral abnormalities (Schubert et al., 2004). Although caution should be exercised in interpreting data generated from transgenic studies, the BIRKO mice might suggest that insulin deficiency is not a crucial contributor to the neural cell death found in the study. Nevertheless, additional work to dissect the role of insulin deficiency from the confounding hyperglycemia in STZ-induced diabetic animals (e.g. use of intracerebroventricular administration of insulin to ameliorate the behavioral abnormality and its associated pathology) would be needed to clarify the point.

In summary, we demonstrated that apoptosis in the frontal cortex and hippocampus were associated with the depressive phenotype of comorbid diabetic animals and that a fission-dominant expression of mitochondrial dynamic genes and proteins played a role in the hyperglycemia-induced neural cell death *in vivo*. The study provides new insight into the disease mechanism and suggests developing novel therapeutics aiming to modulate mitochondrial dynamics to treat comorbid depression with diabetes that is refractory to the current antidepressant treatment.

## MATERIALS AND METHODS

### Animals

Male Kunming mice (8-weeks old, 38.50±1.51 g), a widely used laboratory mouse strain in China, were obtained from the Laboratory Animal Center at Shantou University Medical College. The animals were housed under standard conditions (24±1°C, humidity 60±5%, 12-hour light/12-hour dark cycle) with food and water *ad libitum*. All experimental procedures were conducted in accordance with the guidelines published in the Ministry of Science and Technology of the People’s Republic of China for Care and Use of Laboratory Animals and approved by the Animal Care and Welfare Committee of Shantou University Medical College. Every effort was made to minimize the number of animals used and their suffering.

### Induction of diabetes and blood glucose measurement

Mice were acclimated for 1 week before the start of experiments and were then randomized into two groups. The diabetes group was injected intraperitoneally with STZ (Sigma, USA) dissolved in 0.01 M citrate buffer at pH 4.2. A total of 200 mg STZ/kg body weight was dosed on three separate days, with day 1 at 100 mg/kg, day 3 at 50 mg/kg and day 4 at 50 mg/kg. The control group was injected with the same volume of the citrate buffer. Mice were fasted overnight prior to each injection and the fasting was continued for 90 minutes after the injection.

Blood droplets were obtained by tail nicking at day 0 (before the STZ injection), days 4 and 7 after the STZ injection, and once a week thereafter. Non-fasting blood glucose was measured at 9–10 AM with the Glucotrend monitor and glucose test strips (Roche Diagnostics, Switzerland; detection range 0.6–33.3 mmol/l). Hyperglycemia was defined as the blood glucose level at or higher than 16.7 mmol/l. Body weight was measured weekly. The 24-hour food and water intakes were assessed on day 38.

### Behavioral tests

Behavioral tests were performed in the eighth week after the STZ treatment. The tests were administered at least 1 day apart.

OFT was carried out in a black pixel glass box (278 mm length×236 mm width×300 mm height) illuminated by a 60 W bulb. The floor of the box was outlined by nine squares (three squares long and three squares wide). The squares were used to indicate the location of animals during the test: eight squares were aligned with the edge of the walls (the peripheral area) and one square was inside the outer squares (the center area). Mice were placed gently in the center of the test field and allowed to freely explore for 5 minutes. Four mice were simultaneously tested in individual boxes and movements videotaped. The test area was wiped clean with 70% alcohol between tests. The number of times standing (rearing), the total, central and peripheral distances traveled, and the duration of time spent in the central and peripheral areas were recorded by a video camera mounted on the top of the box. The video tracking data were analyzed by the behavioral analysis system and public domain image processing and analysis program (Mobiledatum, China).

In FST, mice were placed individually into glass cylinders (300 mm height, 200 mm diameter, Mobiledatum, China) containing 200 mm of water maintained at 23–25°C. The immobility was scored when animals were passively floating without movements other than those necessary for keeping the head above water. Swimming was defined as horizontal movements other than passive floating. The test was run and videotaped for the last 5 minutes of the 6 minutes testing period.

In TST, the duration of immobility was quantified using an automated TST device (Video Analysis System for Animal Behaviors, Mobiledatum, China). The base of the mouse tail was aligned with and taped to the bottom of a centrally located, vertical, flat, metal bar that was connected to a force transducer which recorded the time the mouse spent in an immobile state. Mice were suspended for a total of 5 minutes.

### Tissue collection

At the end of the 8-week study, the trunk blood was collected from each mouse, which was then sacrificed by decapitation. Blood samples were centrifuged for 20 minutes at 3000 ***g*** to obtain sera and stored at −80°C until use. Each mouse brain was quickly removed and dissected on ice. One half of the brain (sagittally cut) was treated for immunohistochemical studies. Briefly, brain tissues were immersed successively in 4% paraformaldehyde for 6 hours, 15% saccharose in 0.1 M PBS overnight, and then 30% saccharose in 0.1 M PBS overnight. Brain tissues were then snap-frozen in liquid nitrogen and stored at −80°C until use. Another half of the brain was immediately snap-frozen in liquid nitrogen and stored at −80°C for PCR and western blot analyses.

### BDNF measurement

The serum BDNF concentration was measured using the mouse brain-derived neurotropic factor ELISA kit (R&D Systems, USA) following the manufacturer’s instruction.

### Quantitative PCR (qPCR) measurement

The frontal cortex and hippocampus were dissected and stored in the RNA preservation solution (Solarbio, China) at −20°C. Total cellular RNAs were extracted using the RNAiso Plus kit (Takara, Japan) according to the manufacturer’s instruction. The RNA purity was confirmed by the OD260/280 nm absorption ratio exceeding 1.8. Total RNAs (1 μg) were reverse transcribed using the transcriptor first-strand cDNA synthesis kit (PrimeScript RT Reagent Kit with gDNA Eraser, Takara, Japan) and qPCR was performed using the SYBR^®^ Premix Ex Taq™ II kit (Tli RNaseH Plus, Takara, Japan). Primers used were as follows (5′–3′): Mfn1 (forward: ATTGGGGAGGTGCTGTCTC; reverse: TTCGGTCATAAGGTAGGCTTT), Mfn2 (forward: AGATGTCCCTGCTCTTTTCTC; reverse: TGTGTTCCTGTGGGTGTCTT), Opa1 (forward: GATGACACGCTCTCCAGTGAAG; reverse: CTCGGGGCTAACAGTACAACC), Drp1 (forward: CGGTTCCCTAAACTTCACGA; reverse: GCACCATTTCATTTGTCACG), Fis1 (forward: AAGTATGTGCGAGGGCTGTT; reverse: GGCAGAGAGCAGGTGAGG) and β-actin (forward: CTGTCCCTGTATGCCTCT; reverse: ATGTCACGCACGATTTCC). qPCR reactions were performed in the 7500 Fast Real-Time PCR System (Applied Biosystems, USA) under conditions of 95°C for 30 seconds, followed by 40 cycles at 95°C for 3 seconds, 60°C for 30 seconds. Quantification of the relative mRNA amount was performed using the ΔCt method with the 7500 Software v2.0.6 (Applied Biosystems, USA).

### Western blotting

10% tissue homogenates from the frontal cortex and hippocampus were prepared in lysis buffer, centrifuged at 13,500 ***g*** for 30 minutes at 4°C. Protein concentrations of each sample were determined by the BCA protein assay kit with BSA standards. 60 μg of total proteins were separated on 10% SDS-PAGE and transferred to nitrocellulose membranes. The membranes were incubated for 1 hour with 5% dry skim milk in TBST buffer at room temperature to block nonspecific binding. Membranes were then incubated with primary antibodies against caspase-3 at a dilution of 1:1000 (the rabbit anti-mouse cleaved caspase-3 mAb, Cell Signaling Technology, USA), Bcl-2 at 1:1000 (the rabbit anti-mouse Bcl-2 mAb, Cell Signaling Technology, USA), Mfn2 at 1:1000 (the rabbit anti-mouse mitofusin-2 mAb, Cell Signaling Technology, USA), Drp1 at 1:1000 (the rabbit anti-mouse Drp1 mAb, Cell Signaling Technology, USA), β-actin at 1:2000 (ZS-Bio, China) overnight at 4°C. Subsequently, membranes were incubated with alkaline-phosphatase-conjugated secondary antibodies for 1 hour at room temperature. Bands were visualized using the chromogenic substrate 5-bromo-4-chloro-3-indolyl phosphate in the presence of nitroblue tetrazolium (SIGMA, USA). The band density was scanned and analyzed using the Odyssey Two-Color Infrared Imaging System (LI-COR, USA).

### TUNEL and DAPI staining

Frozen brains were embedded in the OCT compound (SAKURA, USA), cut into 5-μm-thick sections and processed for TUNEL staining using the *in situ* cell death detection fluorescein kit (Roche Diagnostics, Switzerland) according to the manufacturer’s protocol. Briefly, sections were fixed in 4% paraformaldehyde in PBS for 20 minutes at room temperature and permeabilized in 0.1% sodium citrate containing 0.1% Triton X-100 for 2 minutes on ice. Sections were then incubated with 50 μl TUNEL reaction mixture and incubated in a humidified atmosphere for 60 minutes at 37°C in the dark, followed by three washes with PBS. For counterstaining, sections were incubated with DAPI (Vectashield, USA) for 5 minutes at room temperature. Glass coverslips were mounted on glass slides with mounting media. A DAPI filter was used to detect the DAPI staining (blue color) and an FITC filter was used to detect TUNEL staining (green color). TUNEL-positive and DAPI-positive staining patterns were acquired by the Olympus f1000 microscope (Olympus, Japan).

### Quantification of apoptotic cells

Eight sections from the frontal cortex and hippocampus of STZ-treated mice and the corresponding sections of control mice were examined. TUNEL-positive cells were counted in each section, defined as the ones labeled by both fluorescein and DAPI. The percentage of TUNEL-positive cells represents the sum of double-positive cells over DAPI-positive cells of all sections examined in each brain region. The value reported in the Results section was the average of six animals from each group.

### Statistical analysis

Student’s *t*-test was used for group comparisons within experiments. Data were expressed as the mean *±* standard error of the mean (s.e.m.). The bivariate correlation analysis of Spearman’s rank test was used to analyze the association between apoptotic parameters and behavioral test parameters. *P*<0.05 was considered statistically significant.
